# CXR-LT 2026 Challenge: Multi-Center Long-Tailed and Zero Shot Chest X-ray Classification

**Published:** 2026-04-16

**Authors:** Hexin Dong, Yi Lin, Pengyu Zhou, Fengnian Zhao, Alan Clint Legasto, Juno Cho, Dohui Kim, Justin Namuk Kim, Mingeon Kim, Sunwoo Kwak, Gabriel Moyà-Alcover, Ky Trung Nguyen, Thanh-Huy Nguyen, Ha-Hieu Pham, Huy-Hieu Pham, Huy Le Pham, Nikhileswara Rao Sulake, Aina Tur-Serrano, Ruichi Zhang, Ang Zu, Adam E. Flanders, Zhiyong Lu, Ronald M. Summers, Mingquan Lin, Hao Chen, Yuzhe Yang, George Shih, Yifan Peng

**Affiliations:** 1 Department of Population Health Sciences, Weill Cornell Medicine, New York, USA; 2 Department of Radiology, Fuwai Hospital, National Center for Cardiovascular Diseases, Chinese Academy of Medical Sciences and Peking Union Medical College, Beijing, China; 3 Department of Radiology, West China School of Medicine, Sichuan University, Sichuan University Affiliated Chengdu Second People’s Hospital, Chengdu Second People’s Hospital, Chengdu, China; 4 Department of Radiology, Weill Cornell Medicine, New York, USA; 5 School of Electrical Engineering, Korea Advanced Institute of Science and Technology, Daejeon, South Korea; 6 Gwangju Institute of Science and Technology, Gwangju, South Korea; 7 Department of Biomedical Engineering, Case Western Reserve University, Ohio, USA; 8 School of Electrical and Computer Engineering, Cornell Tech, New York, USA; 9 Department of Mathematics and Computer Science, Universitat de les Illes Balears, Palma de Mallorca, Spain; 10 School of Computer Science and Engineering, VNU-HCM International University, Ho Chi Minh City, Vietnam; 11 Vietnam National University, Ho Chi Minh City, Vietnam; 12 School of Computer Science, Carnegie Mellon University, Pittsburgh, USA; 13 VNU-HCM University of Science, Ho Chi Minh City, Vietnam; 14 VinUni-Illinois Smart Health Center, VinUniversity, Hanoi, Vietnam; 15 College of Engineering & Computer Science, VinUniversity, Hanoi, Vietnam; 16 Department of Computer Science and Engineering, Rajiv Gandhi University of Knowledge Technologies, Nuzvid, India; 17 School of Informatics, Xiamen University, Xiamen, China; 18 Department of Radiology, Thomas Jefferson University, Pittsburgh, USA; 19 National Library of Medicine, National Institutes of Health, Bethesda, USA; 20 Clinical Center, National Institutes of Health, Bethesda, USA; 21 Department of Surgery, University of Minnesota Twin Cities, Minneapolis, USA; 22 Department of Computer Science and Engineering, The Hong Kong University of Science and Technology, Hong Kong, China; 23 Computational Medicine and Computer Science, University of California, Los Angeles, Los Angeles, USA

**Keywords:** Chest X-ray, Long-tailed Learning, Zero-shot Generalization, Multi-center Benchmark

## Abstract

Chest X-ray (CXR) interpretation is hindered by the long-tailed distribution of pathologies and the open-world nature of clinical environments. Existing benchmarks often rely on closed-set classes from a single institution, failing to capture the prevalence of rare diseases or the appearance of novel findings. To address this, we present the CXR-LT challenge. The first event, CXR-LT 2023, established a large-scale benchmark for long-tailed multi-label CXR classification and identified key challenges in rare disease recognition. CXR-LT 2024 further expanded the label space and introduced a zero-shot task to study generalization to unseen findings. Building on the success of CXR-LT 2023 and 2024, this third iteration of the benchmark introduces a multi-center dataset comprising over 145,000 images from PadChest and NIH Chest X-ray datasets. Additionally, all development and test sets in CXR-LT 2026 are annotated by radiologists, providing a more reliable and clinically grounded evaluation than report-derived labels. The challenge defines two core tasks this year: (1) Robust Multi-Label Classification on 30 known classes and (2) Open-World Generalization to 6 unseen (out-of-distribution) rare disease classes. This paper summarizes the overview of the CXR-LT 2026 challenge. We describe the data collection and annotation procedures, analyze solution strategies adopted by participating teams, and evaluate head-versus-tail performance, calibration, and cross-center generalization gaps. Our results show that vision-language foundation models improve both in-distribution and zero-shot performance, but detecting rare findings under multi-center shift remains challenging. Our study provides a foundation for developing and evaluating AI systems in realistic long-tailed and open-world clinical conditions.

## 1. Introduction

Chest X-ray (CXR) is one of the most widely used imaging modalities in clinical practice. However, the distribution of abnormal findings in CXR datasets is highly imbalanced, with a small number of common abnormalities accounting for most observations, whereas many clinically important abnormalities occur rarely [1]. Such an imbalance poses a fundamental challenge for standard deep learning methods, which tend to bias predictions toward frequent classes while underperforming on rare but critical “tail” classes [2].

Although numerous methods have been proposed to address class imbalance, most existing studies focus on algorithmic improvements under fixed datasets [3–6]. In contrast, comparatively little attention has been given to benchmark design that captures the combined challenges of long-tailed distributions, open-world conditions, and real-world data variability. In practice, clinical models must not only recognize common abnormalities, but also generalize to infrequent and previously unseen abnormalities, often across institutions and imaging settings [7].

To advance research in this direction, we established the CXR-LT challenge series. The first edition (CXR-LT 2023 [8, 9]) introduced a benchmark for long-tailed multi-label classification. It provided a large-scale training dataset derived from radiology reports and evaluated model performance across a diverse set of abnormal findings with highly imbalanced prevalence. The primary goal was to identify the limitations of existing deep learning models, particularly their difficulty in accurately recognizing infrequent diseases and handling multi-label predictions. The second event (CXR-LT 2024 [10]) expanded the benchmark in two major directions. First, it significantly increased both the dataset scale and label diversity, comprising over 377,000 CXRs annotated with 45 disease categories, including 19 newly introduced rare findings. Second, it introduced a zero-shot learning task to evaluate the model’s ability to recognize previously unseen abnormalities without direct supervision. This addition explicitly studied open-world settings, where models must handle diseases not observed during training.

Building on these efforts, **CXR-LT 2026** further advances the benchmark to better reflect real-world clinical conditions. First, we introduce a multi-center dataset by combining PadChest [11] and NIH Chest X-ray data [12], enabling cross-center evaluation while addressing recent access restrictions of prior datasets [13]. Second, we improve label quality by using radiologist-annotated development and test sets, providing more reliable ground truth than report-derived labels in previous challenges. Third, we define two complementary tasks that jointly evaluate long-tailed classification of known findings and open-world generalization to unseen findings.

This paper presents a comprehensive overview of the CXR-LT 2026 challenge. We detail the dataset construction and label collection, summarize methods developed by participating teams, and provide in-depth performance analyses, including head-to-tail behavior, robustness under input perturbations, and cross-center generalization. The results highlight persistent challenges in long-tailed and open-world CXR analysis and offer insights for developing more robust and clinically applicable AI systems.

## 2. Challenge description

### 2.1. Task description

#### 2.1.1. Task 1: Long-Tailed Multi-Label Classification

The first task evaluates models’ ability to recognize common CXR findings under a long-tailed class distribution. In this task, participants are required to predict the presence or absence of 30 “seen” disease categories. The label set was constructed based on two criteria. First, we retained 20 classes from previous CXR-LT editions to ensure longitudinal consistency. Second, we added 10 additional findings from the PadChest dataset to better capture clinically common abnormalities. The final label set, therefore, reflects a broad and realistic spectrum of thoracic findings: (1) Alveolar Pattern, (2) Aortic Atheromatosis, (3) Aortic Elongation, (4) Atelectasis, (5) Azygos Lobe, (6) Bronchiectasis, (7) Cardiomegaly, (8) Calcified Densities, (9) Central Venous Catheter, (10) Emphysema, (11) Fracture, (12) Hemidiaphragm Elevation, (13) Hernia, (14) Hydropneumothorax, (15) Hyperinflated Lung, (16) Hypoexpansion, (17) Interstitial Pattern, (18) Kyphosis, (19) Mass, (20) Nodule, (21) Normal, (22) Pleural Effusion, (23) Pleural Thickening, (24) Pneumothorax, (25) Pneumoperitoneum, (26) Sternotomy, (27) Subcutaneous Emphysema, (28) Support Devices, (29) Vascular Hilar Enlargement, (30) Vertebral Degenerative Changes.

These categories cover structural abnormalities, pulmonary diseases, and medical devices, spanning both frequent and infrequent findings. Models were trained using the large-scale PadChest training dataset with report-derived labels. Evaluation was conducted on the radiologist-annotated PadChest-GR dataset [14] to ensure a more reliable assessment compared to prior editions.

#### 2.1.2. Task 2: Open-World Generalization

The second task evaluates models’ ability to recognize previously unseen disease categories without explicit supervision. The label set was derived from PadChest-GR and built upon the out-of-distribution (OOD) taxonomy introduced in Task 3 of the CXR-LT 2024. To maintain comparability, we retained the same five OOD categories and introduced one additional finding, Goiter. The final set includes: (1) Adenopathy, (2) Bulla, (3) Goiter, (4) Infarction, (5) Osteopenia, (6) Scoliosis.

During training, models used the same dataset as in Task 1 but did not receive labels for these six findings. The goal is to assess whether models can leverage learned visual representations to generalize to new disease categories. This setup reflects a realistic clinical deployment in which models may encounter abnormalities that were not explicitly annotated during training.

Evaluation was conducted on both an **internal** test set from PadChest-GR and an **external** test set from the NIH ChestX-ray dataset. The inclusion of external data enables assessment under cross-center distribution shifts, further testing model robustness. Compared with Task 1, this task is substantially more challenging, as it requires both generalization to unseen classes and stability across different data sources.

### 2.2. Dataset description

The CXR-LT 2026 dataset was constructed from two publicly available sources: PadChest [11] and NIH ChestX-ray [12]. It is designed to support both large-scale training under weak supervision and rigorous evaluation under clinically realistic conditions, including cross-center variability (Table 1).

#### Training data (PadChest).

PadChest serves as the primary data source for model training. We use 142,928 CXR images, each paired with a Spanish radiology report. Disease labels were automatically extracted from these reports, resulting in a large-scale dataset with weak supervision. This setup reflects real-world conditions, where annotations are available at scale but may be noisy.

#### Internal test (PadChest-GR).

To provide a reliable evaluation, we use PadChest-GR [14], a radiologist-annotated subset of PadChest. In this dataset, multiple radiologists provided image-level annotations for each case. From this subset, we selected 3,020 frontal CXRs that contain at least one of the target disease findings. These images were split into development and test sets using stratified sampling to preserve the long-tailed class distribution.

#### External test (NIH ChestX-ray).

To evaluate generalization, we constructed an additional test set from the NIH ChestX-ray dataset. This dataset differs from PadChest in acquisition protocols, patient populations, and labeling characteristics, introducing a natural distribution shift. It therefore enables evaluation of model robustness across institutions.

### 2.3. Label collection

Label collection plays a central role in the CXR-LT 2026 benchmark. The dataset follows a two-stage annotation strategy that combines large-scale weak supervision for model development with expert-level annotations for evaluation.

For the **training set**, labels were automatically extracted from radiology reports associated with PadChest images. These reports contain detailed descriptions of abnormal findings written by radiologists. Parsing these reports enables scalable label generation, producing a large dataset suitable for model training, albeit with inherent noise typical of weak supervision.

For the **development and test sets**, we used manual radiologist annotations to ensure high-quality evaluation. All images in the PadChest-GR subset were annotated at the image level by multiple radiologists [14], providing a strong reference standard.

For the **external test set** constructed from the NIH ChestX-ray dataset, a stricter annotation protocol was applied. Candidate images were first selected based on the potential presence of rare findings. Each image was then independently reviewed by three radiologists (P.Z., F.Z., A.C.L.). Only cases with agreement from at least 2 radiologists were included in the final dataset, ensuring consistent and high-quality annotations.

### 2.4. Metric

CXR-LT 2026 focuses on multi-label classification under severe class imbalance. In this setting, standard evaluation metrics such as AUROC (Area Under the Receiver Operating Characteristic Curve) can be misleading because performance is heavily influenced by the large number of negative samples in long-tailed datasets. To address this issue, we use mean Average Precision (mAP) as the primary evaluation metric [15]. Specifically, we computed the average precision independently for each class and reported the macro-average across all classes as the final score. This metric evaluates the ranking quality of predicted probabilities across all decision thresholds and provides a more reliable measure of performance in long-tailed multi-label settings.

In addition to mAP, we report several auxiliary metrics to provide complementary insights into model behavior: (1) mean AUROC (mAUROC) to measure ranking performance, (2) mean F1 score (mF1), mean precision, and mean recall, computed using a fixed decision threshold, and (3) Expected Calibration Error (ECE) to assess the reliability of predicted probabilities. These auxiliary metrics are presented on the leaderboard, but only mAP is used for final ranking.

### 2.5. Challenge timeline

The CXR-LT 2026 challenge was organized in a staged timeline to ensure fair evaluation while supporting iterative model development.

#### Training phase (Nov 24, 2025).

The training set was released, allowing participants to explore the dataset, design models, and develop baseline approaches. Teams were required to register for the challenge to obtain access to the data.

#### Development phase (Dec 1, 2025 - Jan 19, 2026).

The development set was made available for iterative evaluation. Participants could submit predictions and access immediate feedback via the online CondaBench platform^1^. This phase enabled performance analysis, error diagnosis, and model refinement.

#### Final evaluation phase (Jan 20, 2026 – Feb 4, 2026).

The final test set was released under ‘blind test’ rules. Specifically, participants were allowed to submit up to 5 valid predictions, without access to ground-truth labels or intermediate results. All submissions were evaluated centrally.

### 2.6. Evaluation protocol

Final rankings were determined based on the primary metric (mAP). Secondary metrics (i.e., mAUC, mF1, ECE, mean precision, and mean recall) were reported for additional insights and used only to break ties when teams have the same mAP scores.

To ensure fairness and reproducibility, the challenge enforced the following guidelines. Participants were **allowed** to use publicly available pretrained models, including large vision or vision–language foundation models. Participants were **not allowed** to use the test data during training or to use external annotations that overlap with the evaluation sets. These rules ensured that all methods were evaluated under consistent conditions while still allowing participants to explore advanced model architectures.

In addition to the primary evaluation, we further considered an auxiliary robustness analysis to assess model sensitivity to input variations. Specifically, test-time perturbations were applied to the evaluation images using standard augmentation operations (e.g., random flipping and intensity variations). Models were evaluated on both the original and perturbed inputs using the same metrics and protocols. This additional analysis is intended to provide complementary insights into model stability under realistic variations in clinical imaging. It does not affect the official ranking, which is determined solely based on performance on the original test set.

## 3. Results

### 3.1. Team participation across stages

A total of 72 teams initially registered and obtained access to the training data. During the development phase, 23 teams submitted predictions to the public leaderboard, resulting in 2,475 submissions in total. This high submission volume reflects sustained engagement and iterative experimentation throughout model development.

**Figure 2:**
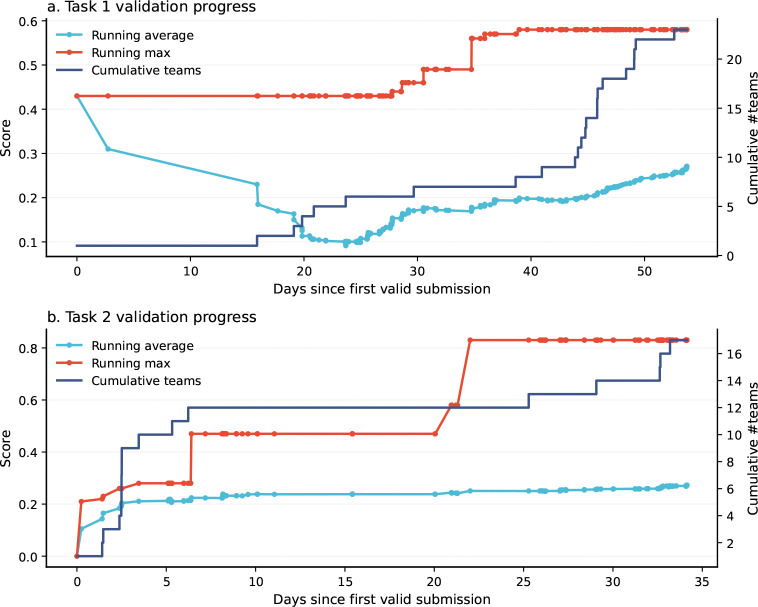
Validation progress over time for Task 1 and Task 2.

As shown in Figure 2, model performance improved steadily over time. The running maximum score exhibits several distinct jumps, indicating moments when new methodological advances or refinements led to notable performance gains. In contrast, the running average score increases more gradually, suggesting consistent incremental improvements across participating teams. In addition, the number of active teams increased during the later stages of the development phase, reflecting growing engagement as participants refined their approaches. Similar trends are observed in both tasks, although performance improvements in Task 2 appear more abrupt, highlighting the greater difficulty of the open-world setting.

At the final evaluation stage, 21 teams submitted complete test-set predictions for official ranking. Following the release of final results, all eligible teams were required to submit technical reports describing their methodology. Only teams that completed this final submission were included in the official challenge summary.

### 3.2. Participating methods

Table 2 summarizes the top-performing methods for both Task 1 and Task 2. For each team, we report key components of their approach, including image resolution, model backbone, training strategy, pretraining dataset, and inference strategy. A clear methodological distinction emerges between the two tasks. For Task 1, most methods rely on convolutional neural networks or transformers combined with techniques such as loss reweighting or sampling strategies to mitigate the effects of long-tailed class distributions. In contrast, Task 2 is dominated by vision-language models, which leverage joint image–text representations for open-world generalization. These approaches typically employ prompt-based inference or metric-based matching strategies.

#### Team A: CVMAIL × MIHL.

This team participated in both Task 1 (long-tailed multi-label classification) and Task 2 (zero-shot OOD recognition) of the CXR-LT 2026 challenge [16]. For Task 1, they develop an imbalance-aware multi-label learning framework based on ConvNeXtV2-Base [17]. To mitigate extreme class imbalance, they adopt a Distribution-Balanced loss with effective-number reweighting and a positive logit-margin adjustment to enhance tail recognition while maintaining stability on head classes. Class-Aware Sampling further increases exposure to rare findings. During inference, they apply test-time augmentation and weighted checkpoint ensembling, followed by a lightweight normal-gating refinement to suppress spurious abnormal predictions. For Task 2, they employ WhyXrayCLIP [18], a CXR-specific vision-language model built on top of OpenCLIP ViT-L/14 [19]. Zero-shot recognition is formulated as image–text similarity matching in a shared embedding space, where unseen disease categories are represented using textual prompts. Prompt ensembling improves robustness to wording variations, and no supervised OOD labels are used during training. This unified framework addresses supervision scarcity through imbalance-aware optimization for in-distribution learning and contrastive vision-language alignment for zero-shot generalization.

#### Team B: Cool Peace.

This team proposed two independent models for CXR-LT classification: one for Task 1 and one for Task 2 [20]. For multi-label classification (Task 1), the team trained an EfficientNet Router to predict which projection an input image belongs to. Based on this prediction, the input image is fed into a corresponding projection-aware model, each trained only on a single projection. The final logit of each branch is obtained by ensembling three model outputs. Each ensemble component uses modern visual encoder architectures, such as ConvNeXt, Class Attention Image Transformer (CaiT), Shifted-Window (Swin) Transformer [21], and their hybrids [22]. The team adopted strong data augmentation, including rotation, horizontal flipping, scaling, and shifting, to enhance robustness. Test-Time Augmentation (TTA) was also applied at the inference stage. For zero-shot classification (Task 2), the team utilized CheXzero [23] as the base vision-language model. To address the severe long-tailed distribution in the PadChest dataset, the architecture was expanded into a dual-branch structure. A classification branch was integrated alongside the original contrastive branch, computing cosine similarities between image and disease description embeddings to generate logits supervised by Asymmetric Loss (ASL) [24]. The optimal loss weighting between the two branches was determined via a proxy Out-Of-Distribution (OOD) validation strategy, curating three distinct experimental sets by splitting the 30 available training classes into 24 seen and 6 unseen classes. Additionally, LLM-generated prompt descriptions were used to extract context-aware text embeddings. During inference, text shuffling and TTA were applied to improve generalizability.

#### Team C: VIU.

This team takes part in Task 1 of the ISBI 2026 CXR-LT challenge [25]. They adopt a lightweight two-phase training framework with a Mixture-of-Expert (MoE) integration strategy to enhance the model performance on the multi-label CXR-LT challenge, specifically in task 1. In the first phase, they fully train a ConvNeXt-B [26] backbone together with a query-based decoder classification head [27]. In the second phase, they freeze most backbone parameters and only train their last layers, together with the classification head. Most importantly, they further incorporate MoE into the architecture by replacing the MLP layer of the last ConvNeXt layers and the decoder head. Through this MoE mechanism, they aim to promote expert specialization across multiple clinical diseases beyond a single shared representation, thereby further improving the model’s generalization. Eventually, they employ an asymmetric loss [24] to mitigate the data imbalance, followed by an auxiliary loss to prevent expert collapse.

#### Team D: Bibimbap-Bueno.

This team utilized the Ark+ backbone [28] for CXR-LT classification in Task 1. Their approach used the Ark+ backbone pretrained on over 700,000 chest X-ray images as the feature extractor. Specifically, the backbone weight of the Swin-Large model [21] was initialized from Ark+ pretrained weights and fine-tuned end-to-end on the challenge dataset. To address the severe class imbalance, they used asymmetric loss (ASL) [24], which downweights easy negatives and focuses learning on rare positive findings in the long tail. Test-time augmentation was implemented via multi-crop evaluation (n=10) using multiple spatial crops and their horizontal flips with prediction averaging.

#### Team E: Nikhil Rao.

This team participated in Task 1. The approach systematically evaluates imbalance-aware loss functions and modern CNN architectures on the CXR-LT 2026 benchmark. The primary strategy employed Label-Distribution-Aware Margin (LDAM) loss [29] with Deferred Re-Weighting (DRW) to improve minority-class margins. Multiple backbones were explored, including ResNet, DenseNet, EfficientFormerV2, and ConvNeXt [26] variants. A two-stage classifier retraining (cRT) [30] strategy was applied to decouple representation learning from classifier optimization. Test-time augmentation (horizontal flips and small rotations) and weighted probability ensembling were further used to enhance robustness. ConvNeXt-Large achieved the strongest single-model performance, while ConvNeXt-Base with cRT+TTA improved ranking metrics. A weighted ensemble of ConvNeXt-Large, ConvNeXt-Base, and EfficientNetV2-S achieved 52% mAP on the development set.

#### Team F: UGIVIA.

This team developed a two-stage framework for CXR-LT Task 1. Their approach first employed a binary DenseNet121 model to distinguish normal from pathological chest X-rays, then a multi-label DenseNet121 to predict specific diseases. Both models used CheXpert- pretrained weights from TorchXRayVision [31], with DenseBlock 3–4 finetuned. To handle long-tailed distributions, the multi-label model included a projection head optimizing a weighted combination of Asymmetric Loss ($\gamma_{neg}=6,\;\gamma_{pos}\;=\;1$) [24] and Multi-Supervised Contrastive Loss (α=1.0,β=1.0, temperature = 0.07) [32]. To address class imbalance, they applied targeted augmentations (rotations and horizontal flips) for classes with fewer than 4,000 samples and multilabel-stratified splitting. During inference, the binary classifier predicted images as normal or diseased, forwarding pathological images to the multi-label model, while normal images were directly labeled normal.

#### Team G: Zuang.

This team proposes a framework for long-tailed multi-label chest X-ray classification and zero-shot recognition of unseen diseases, participating in Task 2 of the CXR-LT challenge. Zero-shot recognition of unseen findings in long-tailed multi-label chest X-ray classification remains a fundamental challenge. To address this, this team proposes a method that combines model fine-tuning with a class-prototype prompting mechanism. Semantic-Aware Vision–Language Pretraining leverages a medical vision–language model to align chest X-ray images with structured disease label representations, enabling generalization to unseen categories [33]. Domain- Adaptive Fine-Tuning adapts the model to the target CXR dataset, enhancing discriminative feature learning while preserving cross-modal semantic knowledge to better handle long-tailed distributions. Finally, Prototype-Based Zero-Shot Inference constructs robust class prototypes from high-confidence samples, providing stable semantic anchors that guide zero-shot prediction for unseen diseases [34]. Extensive experiments validate the effectiveness of our framework, achieving a mAP of 0.2235 and ranking third among all participants.

### 3.3. Primary evaluation results

**Figure 3:**
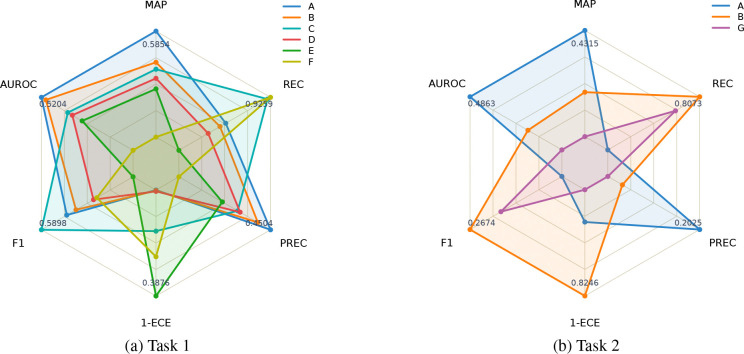
Radar plots summarizing the main results of CXR-LT 2026 for the two challenge tasks. **a**, Task 1 evaluates long-tailed multi-label classification on seen classes. **b**, Task 2 evaluates generalization to unseen rare findings in an open-world setting. The plots compare the leading methods across six evaluation metrics: mAP, AUROC, F1, 1−ECE, precision, and recall. Please note we report 1−ECE as a direction-aligned calibration score, such that higher values indicate better calibration.

The primary results of CXR-LT 2026 are shown in Figure 3 and detailed in Extended Data Table 1. Model performance is primarily evaluated using mean Average Precision (mAP) for ranking, while additional metrics (AUROC, F1 score, ECE, precision, and recall) are reported to provide complementary perspectives on model behavior. Confidence intervals are estimated using 1,000 bootstrap samples.

Task 1 evaluates multi-label classification on 30 known abnormal findings under a long-tailed class distribution. The top-performing method achieves an mAP of 0.5854, with a clear margin over the other submissions. AUROC scores are generally high across teams, indicating that most methods can effectively distinguish positive and negative samples at a ranking level. However, substantial differences appear in threshold-dependent metrics. In particular, some methods achieve higher F1 and recall despite having lower ranking metrics, suggesting that their predictions may operate at different confidence thresholds. This discrepancy indicates that strong ranking performance does not necessarily translate into optimal binary classification outcomes. Calibration performance also varies considerably across teams. Although achieving high AUROC scores, several methods exhibit relatively large ECE, indicating that their predicted probabilities are not well aligned with true outcome frequencies. This limitation is important in clinical settings, where reliable probability estimates are critical for decision support.

Task 2 evaluates model generalization to six unseen disease categories not included in the training data. Compared with Task 1, performance drops substantially across all metrics, reflecting the challenge of recognizing previously unseen abnormalities. The best-performing method achieves an mAP of 0.4315, while the remaining methods show noticeably lower performance. This gap indicates that current models still struggle to generalize beyond the label space seen during training. Consistent with Task 1, ranking-based and threshold-dependent metrics do not always align. For example, some methods achieve higher recall despite lower mAP, suggesting different trade-offs between sensitivity and ranking quality.

Figure 3 also reveals three consistent observations. First, large performance gaps remain between the top-performing methods and the remaining submissions, indicating substantial differences in how models handle long-tailed distributions. Second, ranking-based metrics such as mAP and AUROC do not always align with threshold-dependent metrics such as F1 and recall, suggesting that different modeling strategies favor different operating regimes. Third, performance drops markedly in the open-world setting, highlighting the challenge of recognizing previously unseen abnormalities.

### 3.4. Robustness to test-time perturbations

**Figure 4:**
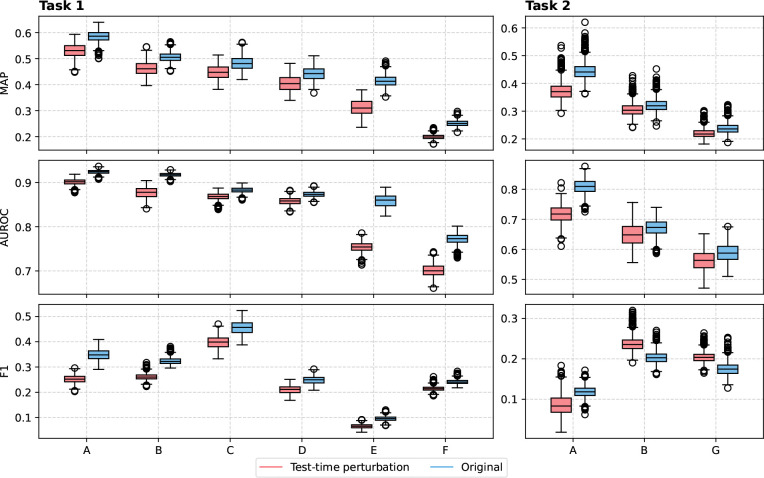
Robustness analysis under test-time perturbations. Each panel compares model performance under test-time perturbations and original test data.

To evaluate the robustness of the models, we conducted an additional analysis under test-time perturbations. Specifically, we applied augmentation-based transformations to the test images and compared model performance against the original evaluation setting. This analysis allows us to examine how sensitive model predictions are to small variations in the input data. Figure 4 illustrates the performance differences across teams and evaluation metrics, and Extended Data Table 2 summarizes the quantitative results for Task 1 and Task 2. Overall, most methods exhibit performance decreases under test-time perturbations. In Task 1, the best-performing method achieves an mAP of 0.5854 under the original setting but drops to 0.5292 with perturbations. Similar declines are observed across other teams, suggesting that even high-performing models remain sensitive to input variations.

The effect is more pronounced for ranking-based metrics (e.g., mAP and AUROC), whereas threshold-dependent metrics (e.g., F1) show greater variability across teams. This observation indicates that perturbations mainly affect the relative ranking rather than fixed decision thresholds. A comparable trend is observed in Task 2. Although baseline performance is lower due to the open-world setting, test-time perturbations still lead to consistent drops across most teams.

### 3.5. Evaluation on held-out subsets

To further examine the stability of model performance, we conducted additional evaluations on held-out subsets constructed from the available data. For Task 1, a subset of 300 cases was sampled from the training data, while for Task 2, a subset of 200 cases was selected from the development set. All models were evaluated on these subsets using the same protocol as in the main evaluation.

**Figure 5:**
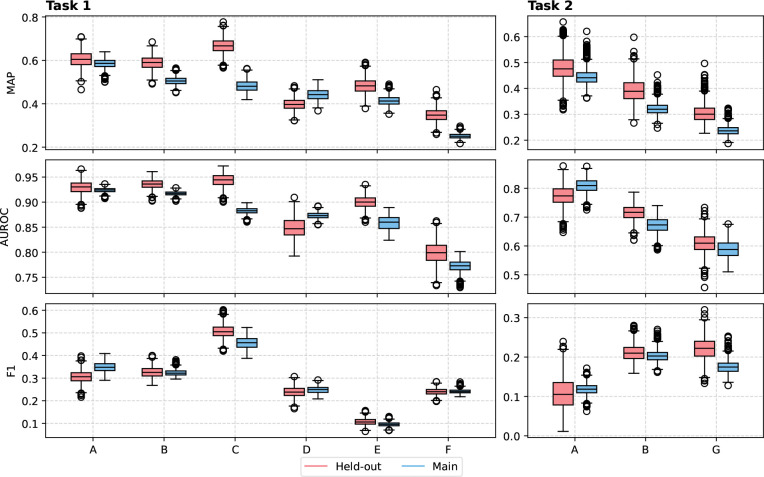
Evaluation on held-out subsets for Task 1 and Task 2. Each panel compares model performance across teams under held-out subsets and the main evaluation. The left column shows results for Task 1, and the right column shows results for Task 2, with mAP, AUROC, and F1 reported as representative metrics. Across most teams, the relative ranking of methods remains consistent between evaluation settings, while overall performance tends to decrease on the held-out subsets.

The results are summarized in Figure 5 and detailed in Extended Data Table 3. Overall, the relative performance of different methods remains largely consistent with the main evaluation. Methods that perform well on the full evaluation set tend to achieve higher scores on the held-out subsets as well, indicating that the ranking of methods is generally stable across different data samples. At the same time, performance on the held-out subsets is typically lower than on the main evaluation set. This trend is consistent across most teams and metrics and may be attributed to differences in data composition and the smaller sample size of the subsets.

We also observe that the impact varies across metrics. Ranking-based metrics such as mAP and AUROC show more consistent trends across evaluation settings, while threshold-dependent metrics such as F1 exhibit greater variability. This suggests that model ranking is relatively robust, whereas threshold-based performance is more sensitive to changes in evaluation data.

### 3.6. Head-to-tail performance

**Figure 6:**
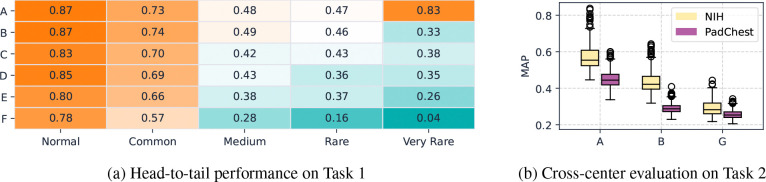
Generalization analysis across disease frequency and clinical centers. a) Head-to-tail performance on Task 1 across disease groups with different occurrence frequencies. b) Cross-center evaluation on Task 2, comparing performance between the NIH ChestX-ray and PadChest subsets.

To better understand model behavior under long-tailed disease distributions, we analyzed performance across disease frequency. Diseases were grouped into five categories: normal cases, common diseases (> 10%), medium-frequency diseases (1% – 10%), rare diseases (0.1% – 1%), and very rare diseases (< 0.1%). As shown in Figure 6a and detailed in Extended Data Table 4, model performance consistently decreases as disease frequency becomes lower. While most methods achieve strong performance on normal and common disease categories, performance drops noticeably for rare and very rare diseases. This trend highlights the persistent challenge of long-tailed recognition in CXR analysis. In particular, the performance gap between common and rare disease groups remains substantial across all teams, suggesting that current methods still struggle to learn and generalize visual patterns for low-frequency abnormalities.

### 3.7. Multi-center generalizaiton

To further examine model cross-center generalization, we analyzed model performance separately on the PadChest and NIH ChestX-ray subsets of Task 2. As shown in Figure 6b and detailed in Extended Data Table 5, all participating methods achieve consistently higher performance on the NIH subset. For example, all three evaluated methods achieve noticeably higher mAP values on NIH compared with PadChest. This performance gap may be attributed to differences in imaging characteristics or data composition between the two datasets.

## 4. Discussion

In this study, we present the CXR-LT 2026 challenge, a multi-center benchmark designed to evaluate long-tailed learning and open-world generalization in chest X-ray analysis. Compared with previous editions, this benchmark introduces radiologist-annotated evaluation sets and cross-center testing, providing a more realistic setting for assessing model performance.

Several key observations emerge from the results. First, although top-performing methods achieve strong ranking performance on common findings, substantial performance gaps remain across teams, particularly under long-tailed distributions. Performance consistently decreases for low-frequency disease categories, indicating that current methods still struggle to capture visual patterns for rare abnormalities. This gap suggests that existing strategies for handling class imbalance, such as reweighting or resampling, remain insufficient.

Second, we observe a clear mismatch between different evaluation metrics. As shown in Figure 3a, ranking-based metrics do not always align with threshold-dependent metrics. Methods that perform well in mAP or AUROC do not necessarily achieve strong classification performance in F1 or recall, suggesting differences in prediction calibration and optimal thresholds. Consistently, many methods also exhibit suboptimal calibration, indicating that predicted probabilities are not always reliable for clinical decision support.

Third, performance drops significantly in the open-world setting (Figure 3b, where models are required to recognize unseen disease categories. While vision-language models improve zero-shot performance, the overall results suggest that generalization to new disease concepts remains limited. This finding highlights the gap between current supervised learning approaches and real-world clinical requirements.

Beyond these main findings, additional analyses provide further insights into model behavior. The robustness experiments show that model predictions remain sensitive to input perturbations (Figure 4), even for top-performing methods. Evaluation on held-out subsets demonstrates that while relative rankings are generally stable, absolute performance can vary across data splits. Multi-center analysis further reveals consistent performance differences across datasets, indicating that domain shifts between institutions remain an important challenge.

## 5. Conclusion

The CXR-LT 2026 benchmark highlights several open challenges for radiology AI, including long-tailed recognition, probability calibration, robustness to data variation, and cross-center generalization. Addressing these issues will likely require improved representation learning, better calibration methods, and approaches that explicitly account for distribution shifts across clinical settings. The benchmark provides a unified testbed for studying these problems under realistic conditions and for developing more robust and clinically applicable models.

## Extended Data

**Extended Data Table 1:** Main results with 95% bootstrap confidence intervals based on 1,000 resampling runs.

Team	mAP	AUROC	F1	ECE	Precision	Recall

Task 1						
A	0.585 (0.540–0.621)	0.926 (0.915–0.932)	0.352 (0.305–0.390)	0.920 (0.912–0.923)	0.590 (0.498–0.618)	0.296 (0.255–0.337)
B	0.483 (0.473–0.543)	0.919 (0.909–0.925)	0.316 (0.303–0.354)	0.918 (0.910–0.921)	0.531 (0.472–0.596)	0.267 (0.255–0.300)
C	0.460 (0.437–0.532)	0.883 (0.870–0.894)	0.450 (0.405–0.499)	0.801 (0.784–0.804)	0.439 (0.383–0.488)	0.505 (0.456–0.574)
D	0.430 (0.396–0.487)	0.875 (0.861–0.886)	0.248 (0.218–0.275)	0.916 (0.908–0.919)	0.450 (0.372–0.481)	0.207 (0.187–0.237)
E	0.395 (0.373–0.458)	0.859 (0.834–0.881)	0.095 (0.077–0.115)	0.612 (0.603–0.614)	0.368 (0.311–0.409)	0.061 (0.047–0.077)
F	0.236 (0.231–0.276)	0.776 (0.746–0.792)	0.235 (0.225–0.262)	0.726 (0.702–0.738)	0.168 (0.161–0.189)	0.520 (0.496–0.569)
Task 2						
A	0.432 (0.390–0.523)	0.807 (0.755–0.850)	0.118 (0.091–0.147)	0.452 (0.433–0.464)	0.267 (0.233–0.322)	0.102 (0.072–0.133)
B	0.311 (0.278–0.380)	0.671 (0.614–0.723)	0.203 (0.178–0.237)	0.175 (0.153–0.199)	0.196 (0.174–0.233)	0.486 (0.394–0.608)
G	0.224 (0.204–0.278)	0.590 (0.538–0.657)	0.174 (0.147–0.206)	0.573 (0.525–0.590)	0.182 (0.157–0.221)	0.385 (0.325–0.464)

**Extended Data Table 2:** Augmentation results with 95% bootstrap confidence intervals based on 1,000 resampling runs.

Team	mAP	AUROC	F1	ECE	Precision	Recall

Task 1						
A	0.529 (0.469–0.579)	0.903 (0.886–0.913)	0.254 (0.220–0.280)	0.920 (0.912–0.923)	0.522 (0.441–0.545)	0.194 (0.170–0.227)
B	0.443 (0.414–0.513)	0.878 (0.854–0.897)	0.256 (0.239–0.289)	0.919 (0.912–0.922)	0.564 (0.477–0.619)	0.202 (0.188–0.232)
C	0.433 (0.399–0.499)	0.870 (0.849–0.883)	0.394 (0.354–0.443)	0.772 (0.754–0.777)	0.367 (0.328–0.420)	0.458 (0.408–0.524)
D	0.393 (0.354–0.454)	0.860 (0.841–0.876)	0.212 (0.181–0.238)	0.916 (0.908–0.918)	0.459 (0.389–0.489)	0.159 (0.136–0.189)
E	0.302 (0.263–0.365)	0.756 (0.731–0.774)	0.065 (0.050–0.083)	0.608 (0.598–0.609)	0.349 (0.274–0.381)	0.040 (0.030–0.053)
F	0.188 (0.183–0.219)	0.698 (0.674–0.728)	0.210 (0.199–0.235)	0.753 (0.730–0.764)	0.154 (0.146–0.173)	0.387 (0.356–0.433)
Task 2						
A	0.360 (0.319–0.447)	0.716 (0.656–0.768)	0.087 (0.042–0.139)	0.457 (0.440–0.470)	0.359 (0.111–0.429)	0.054 (0.024–0.091)
B	0.295 (0.266–0.363)	0.649 (0.582–0.732)	0.236 (0.206–0.284)	0.167 (0.145–0.187)	0.219 (0.193–0.271)	0.536 (0.436–0.664)
G	0.206 (0.191–0.262)	0.563 (0.495–0.624)	0.203 (0.181–0.234)	0.566 (0.527–0.582)	0.174 (0.161–0.205)	0.556 (0.453–0.688)

**Extended Data Table 3:** Evaluation results on held-out subsets with 95% bootstrap confidence intervals based on 1,000 resampling runs. For each team, we report results trained with and without augmentation.

Team	Aug	mAP	mAUC	mF1	mECE	Precision	Recall

Task 1							
A	✗	0.591 (0.539–0.674)	0.928 (0.903–0.953)	0.313 (0.252–0.361)	0.925 (0.916–0.930)	0.475 (0.367–0.545)	0.278 (0.228–0.326)
	✓	0.458 (0.430–0.548)	0.894 (0.872–0.919)	0.260 (0.196–0.300)	0.924 (0.914–0.929)	0.417 (0.317–0.467)	0.220 (0.164–0.262)
B	✗	0.551 (0.532–0.650)	0.935 (0.917–0.953)	0.316 (0.282–0.375)	0.925 (0.916–0.929)	0.487 (0.394–0.559)	0.272 (0.252–0.327)
	✓	0.420 (0.403–0.523)	0.900 (0.872–0.921)	0.233 (0.192–0.287)	0.924 (0.914–0.929)	0.453 (0.305–0.511)	0.189 (0.157–0.243)
C	✗	0.652 (0.602–0.732)	0.945 (0.920–0.965)	0.498 (0.449–0.563)	0.806 (0.790–0.812)	0.419 (0.374–0.494)	0.698 (0.637–0.790)
	✓	0.506 (0.472–0.598)	0.912 (0.881–0.934)	0.402 (0.355–0.462)	0.771 (0.753–0.780)	0.326 (0.286–0.392)	0.585 (0.524–0.674)
D	✗	0.375 (0.348–0.451)	0.838 (0.811–0.889)	0.245 (0.191–0.281)	0.917 (0.908–0.922)	0.345 (0.271–0.403)	0.218 (0.170–0.254)
	✓	0.340 (0.311–0.410)	0.832 (0.805–0.873)	0.219 (0.166–0.252)	0.916 (0.906–0.921)	0.373 (0.268–0.417)	0.185 (0.136–0.219)
E	✓	0.455 (0.415–0.548)	0.900 (0.876–0.923)	0.105 (0.079–0.136)	0.615 (0.606–0.618)	0.283 (0.181–0.323)	0.081 (0.066–0.105)
	✓	0.303 (0.268–0.387)	0.800 (0.767–0.836)	0.064 (0.034–0.093)	0.611 (0.601–0.614)	0.240 (0.115–0.278)	0.043 (0.020–0.068)
F	✗	0.322 (0.293–0.406)	0.798 (0.756–0.842)	0.231 (0.212–0.271)	0.698 (0.667–0.726)	0.155 (0.142–0.185)	0.688 (0.620–0.757)
	✗	0.223 (0.207–0.303)	0.737 (0.688–0.793)	0.207 (0.178–0.246)	0.705 (0.670–0.732)	0.136 (0.118–0.169)	0.542 (0.462–0.630)

Task 2							
A	✗	0.460 (0.376–0.593)	0.776 (0.702–0.838)	0.114 (0.037–0.192)	0.448 (0.428–0.468)	0.442 (0.189–0.500)	0.081 (0.026–0.149)
	✓	0.333 (0.283–0.451)	0.716 (0.638–0.781)	0.000 (0.000–0.000)	0.453 (0.432–0.475)	0.000 (0.000–0.000)	0.000 (0.000–0.000)
B	✗	0.375 (0.305–0.487)	0.716 (0.665–0.768)	0.213 (0.171–0.257)	0.178 (0.144–0.216)	0.224 (0.193–0.259)	0.562 (0.442–0.663)
	✓	0.361 (0.294–0.468)	0.684 (0.617–0.742)	0.233 (0.186–0.280)	0.148 (0.123–0.184)	0.233 (0.197–0.277)	0.549 (0.424–0.661)
G	✗	0.280 (0.248–0.385)	0.608 (0.542–0.674)	0.226 (0.165–0.276)	0.550 (0.535–0.578)	0.259 (0.214–0.307)	0.455 (0.321–0.585)
	✓	0.248 (0.226–0.329)	0.574 (0.510–0.641)	0.253 (0.214–0.287)	0.527 (0.509–0.554)	0.229 (0.207–0.253)	0.660 (0.525–0.784)

**Extended Data Table 4:** Head to Tail Results in Task 1.

Team	Normal	Common	Medium	Rare	Very Rare

A	0.873	0.734	0.484	0.468	0.826
B	0.870	0.736	0.489	0.455	0.328
C	0.835	0.695	0.424	0.435	0.381
D	0.852	0.690	0.429	0.359	0.347
E	0.796	0.661	0.384	0.368	0.258
F	0.780	0.568	0.281	0.157	0.038

**Extended Data Table 5:** Multi-center evaluation with 95% bootstrap confidence intervals (1,000 runs) in Task 2. Results are reported separately on PadChest and NIH subsets.

Team	mAP	mAUC	mF1	mECE	Precision	Recall

PadChest						
A	0.436 (0.371–0.538)	0.767 (0.718–0.815)	0.130 (0.093–0.164)	0.450 (0.434–0.467)	0.313 (0.274–0.354)	0.115 (0.075–0.155)
B	0.272 (0.246–0.340)	0.647 (0.591–0.704)	0.193 (0.162–0.225)	0.146 (0.126–0.173)	0.217 (0.194–0.253)	0.458 (0.368–0.550)
G	0.241 (0.219–0.308)	0.560 (0.512–0.605)	0.189 (0.146–0.227)	0.524 (0.511–0.547)	0.217 (0.188–0.250)	0.410 (0.304–0.510)

NIH ChestX-ray						
A	0.514 (0.486–0.673)	0.840 (0.786–0.892)	0.141 (0.095–0.205)	0.408 (0.365–0.430)	0.250 (0.167–0.400)	0.112 (0.061–0.180)
B	0.385 (0.358–0.538)	0.629 (0.579–0.766)	0.267 (0.225–0.369)	0.228 (0.190–0.271)	0.206 (0.174–0.290)	0.420 (0.342–0.606)
G	0.258 (0.236–0.371)	0.525 (0.457–0.587)	0.169 (0.132–0.236)	0.596 (0.561–0.624)	0.152 (0.116–0.221)	0.443 (0.281–0.517)

**Extended Data Table 6:** Detailed results of the main evaluation for Task 2 across all categories and participating teams, measured by mean Average Precision (mAP).

Category	A	B	C	D	E	F

Normal	0.873	0.870	0.835	0.852	0.796	0.780
Aortic elongation	0.626	0.634	0.616	0.572	0.561	0.467
Cardiomegaly	0.757	0.749	0.719	0.713	0.664	0.650
Pleural effusion	0.820	0.823	0.751	0.785	0.758	0.586
Nodule	0.479	0.459	0.389	0.458	0.309	0.180
Atelectasis	0.533	0.520	0.433	0.497	0.362	0.195
Pleural thickening	0.492	0.517	0.447	0.391	0.321	0.162
Aortic atheromatosis	0.356	0.358	0.211	0.219	0.226	0.178
Support devices	0.965	0.962	0.970	0.940	0.928	0.767
Alveolar pattern	0.590	0.589	0.526	0.585	0.527	0.454
Fracture	0.429	0.449	0.373	0.310	0.273	0.068
Hernia	0.847	0.789	0.802	0.421	0.534	0.205
Emphysema	1.000	0.121	0.255	0.503	0.126	0.036
Azygos lobe	0.946	0.958	0.892	0.012	0.693	0.017
Hydropneumothorax	1.000	0.013	0.003	0.167	0.009	0.008
Kyphosis	0.190	0.200	0.163	0.110	0.211	0.100
Mass	0.129	0.041	0.066	0.068	0.060	0.029
Pneumothorax	0.608	0.143	0.437	0.689	0.076	0.013
Subcutaneous emphysema	0.833	0.583	0.500	0.700	0.129	0.148
Pneumoperitoneo	0.571	0.146	0.200	0.013	0.518	0.006
Vascular hilar enlargement	0.314	0.301	0.296	0.204	0.268	0.162
Vertebral degenerative changes	0.273	0.293	0.239	0.166	0.172	0.160
Hyperinflated lung	0.304	0.307	0.250	0.242	0.259	0.207
Interstitial pattern	0.538	0.580	0.478	0.591	0.469	0.346
Central venous catheter	0.931	0.965	0.954	0.837	0.870	0.384
Hypoexpansion	0.368	0.457	0.330	0.267	0.304	0.153
Bronchiectasis	0.155	0.182	0.113	0.174	0.120	0.096
Hemidiaphragm elevation	0.563	0.535	0.492	0.403	0.410	0.276
Sternotomy	0.899	0.872	0.924	0.931	0.870	0.239
Calcified densities	0.172	0.063	0.130	0.072	0.028	0.016

**Mean**	**0.585**	**0.483**	**0.460**	**0.430**	**0.395**	**0.236**

**Extended Data Table 7:** Detailed results of the main evaluation for Task 2 across all categories and participating teams, measured by mean Average Precision (mAP).

Category	A	B	G

Scoliosis	0.878	0.737	0.606
Osteopenia	0.127	0.069	0.079
Bulla	0.640	0.448	0.253
Infarction	0.023	0.012	0.020
Adenopathy	0.305	0.419	0.291
Goiter	0.617	0.178	0.091

**Mean**	**0.432**	**0.311**	**0.223**

## Figures and Tables

**Figure 1:**
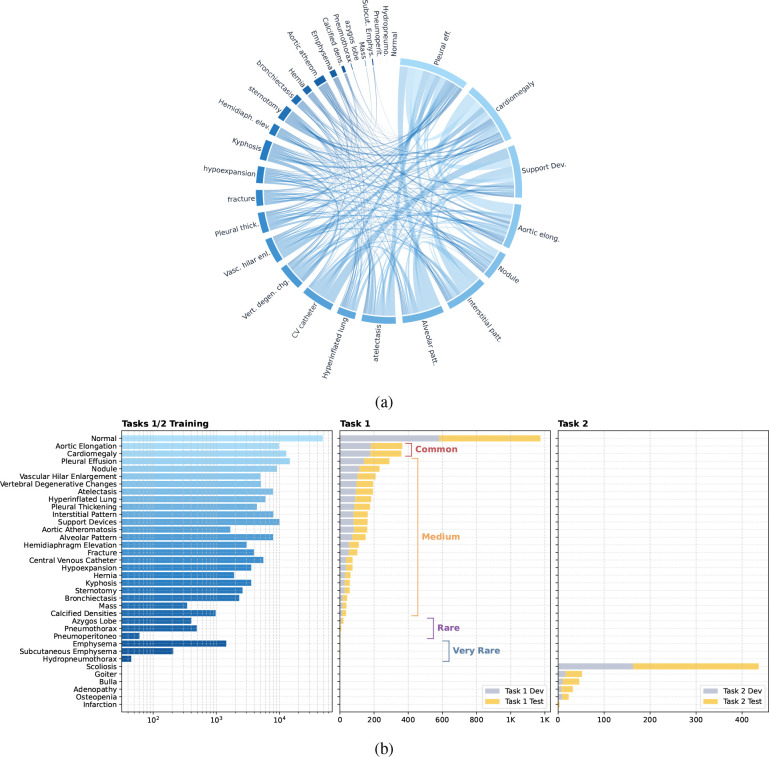
The dataset of CXR-LT 2026. (a) The co-occurrence of labels in the training set. (b) The label distributions in the training set, the development and test sets of Task 1, and the development and test sets of Task 2.

**Table 1:** Overview of the CXR-LT 2026 dataset.

Tasks	Dataset	Source	# CXRs	Curation

1	Training	PadChest	142,928	From reports
	Development	PadChest-GR	1,620	By radiologists
	Test	PadChest-GR	1,400	By radiologists

2	Training	PadChest	142,928	From reports
	Development	PadChest-GR	200	By radiologists
	Internal Test	PadChest-GR	305	By radiologists
	External Test	NIH Chest X-ray	79	By radiologists

**Table 2:** Summary of the top-performing methods for Task 1 and Task 2.

Team	Institution	Image Resolution	Backbone	Strategy	Pretraining	Inference Strategy

Task 1						
A	VinUni-Illinois Smart Health Center	512	ConvNextv2-Base	Distribution-Balanced loss	MIMIC-CXR	TTA, Model ensemble
B	Korea Advanced Institute of Science and Technology	512	ConvNeXtV2, SwinV2-T	-	PCAM pretraining	TTA
C	Vietnam National University	384	ConvNeXt	Asymmetric Loss, Auxiliary Loss	ImageNet	TTA
D	Cornell University	768	Swin-L	Asymmetric loss	Ark+Checkpoint	TTA
E	Rajiv Gandhi University of Knowledge Technologies	512	ConvNext-Large	LDAM, class-balanced sampling	ImageNet	TTA
F	Universitat de les Illes Balears	224	DenseNet121	Asymmetric Loss, Multi-Supervised Contrastive Loss	CheXpert	

Task 2						
A	VinUni-Illinois Smart Health Center	224	OpenCLIP ViT-L/14	Prompt engmeering& prompt ensembling	MIMIC-CXR	Metric-based inference
B	Korea Advanced Institute of Science and Technology	224	CheXzero	Asymmetric Loss	MIMIC-CXR, CheXpert	TTA, Prompt ensembling
G	Xiamen University	224	MedKlip, Resnet50, ClinicalBERT	Class-balanced loss	MIMIC-CXR, CheXpert	Prototype Prompting

## Footnotes

1 Task 1: https://www.codabench.org/competitions/11470/; Task 2: https://www.codabench.org/competitions/11471/.
